# Supplementary material for: A set of multi-entry identification keys to African frugivorous flies (Diptera, Tephritidae)
Source: Zookeys. 2014 Jul 24;(428):97–108. doi: 10.3897/zookeys.428.7366 (PMC4143993; doi:10.3897/zookeys.428.7366)
Supplement: Supplementary material 6 — Key to Ceratitis [file zookeys-428-097-s006.zip › SF6_ZooKeys_key to Ceratitis/key/SF6_ZooKeys_key to Ceratitis/Media/Html/Ceratitis barbata.htm]

Ceratitis (Pterandrus) barbata, n


***Ceratitis*** **(*Pterandrus*) *barbata* De Meyer & Freidberg**

Body
length: 5.68 (5.30-6.40) mm; wing length: 6.21 (5.75-6.50) mm.

Male

Head:
Antenna yellow. First flagellomere 2-3 times as long as pedicel. Arista with
short rays; ventral rays shorter and sparser than dorsal rays, especially
basally. Frons yellow, sometimes with darker center; with short scattered
setulae of same color as frons. Frontal setae well developed. Face white, gena
with dark patch. Genal seta and setulae dark, well developed.

Thorax: Postpronotal
lobe yellowish white; without spot. Scutal pattern ground color gray with
orange tinge; with streaks and darker markings but without distinct spots or
clearly defined stripes except prescutellar white markings merged. Scapular
setae dark. Scutellum yellowish white, basally without spots, apically with
three separate black spots, extending to basal half. Anepisternum along ventral
margin yellowish brown; setulae pale.

Legs:
Yellow except where otherwise noted; setation typical for subgenus, mainly dark.
Foreleg: femur brown; posteriorly with poorly developed bush of dispersed long
dark setulae along entire length, posterodorsal setulae longer; ventral setae
dark. Midleg: femur largely brown except distally pale; ventrally with dark
feathering along entire length, basally developed into dense tuft of dark
setulae; anteroventrally with short but conspicuous dark setulae except on pale
part; tibia broadened; largely yellowish brown with extremities pale, with weak
silvery shine when viewed from certain angle; with black feathering dorsally
along distal 0.9 and ventrally along distal 0.6, anteriorly with short to
moderately long setulae, second row with shorter setulae present anterior to
dorsal feathering. Hindleg: femur dark brown on dorsal half except distally;
with longer setulae dorsally and ventrally at apical 0.25.

Wing:
Bands yellowish brown. Interruption between marginal and discal bands narrow,
often incomplete; cubital band free; medial band short and narrow, sometimes
obscured; crossvein R-M just proximal to middle of discal cell. Apex of vein R_1_
distal to level of crossvein R-M. Crossvein DM-Cu variable.

Abdomen:
Mostly yellow. Border between tergites 1 and 2 with orange-brown patches.
Tergites 2 and 4 with pale gray band occupying almost entire tergite, at most
narrowly yellow anteriorly. Tergite 3 with distinct brownish black band along
posterior half. Tergite 5 anteriorly with two brown spots, posterior margin
narrowly brownish. Male epandrium in lateral view with posterior lobe of
lateral surstylus elongate, tip slightly curved.

Female

As
male except following characters. Frons sometimes completely pale. Anepisternum
at most with few dark setulae along ventral margin posteriorly. Legs without
feathering; orange-brown; forefemur posteroventrally with dark setulae.
Oviscape at least as long as preabdomen. Aculeus very long, at least 11 times
as long as wide; tip without distinct apical indentation, notch weak or tip
almost straight; lateral margin weakly sinuous.

(Description
after De Meyer & Freidberg, 2006)
